# Appraising the Causal Association of Plasma Homocysteine Levels With Atrial Fibrillation Risk: A Two-Sample Mendelian Randomization Study

**DOI:** 10.3389/fgene.2021.619536

**Published:** 2021-05-26

**Authors:** Songzan Chen, Fangkun Yang, Tian Xu, Yao Wang, Kaijie Zhang, Guosheng Fu, Wenbin Zhang

**Affiliations:** ^1^Key Laboratory of Biotherapy of Zhejiang Province, Department of Cardiology, Sir Run Run Shaw Hospital, School of Medicine, Zhejiang University, Hangzhou, China; ^2^Department of Cardiology, Second Affiliated Hospital, School of Medicine, Zhejiang University, Hangzhou, China

**Keywords:** homocysteine, atrial fibrillation, Mendelian randomization, causal association, genome-wide association study

## Abstract

**Background:**

Although several observational studies have suggested an association of elevated plasma homocysteine (Hcy) levels with increased risk of atrial fibrillation (AF), it remains unclear whether this association reflects causality. In this study, we aimed to investigate the causal association of plasma Hcy levels with AF risk.

**Methods:**

A two-sample Mendelian randomization (MR) study was designed to investigate the causal association of Hcy with AF. Summary data on association of single nucleotide polymorphisms (SNPs) with Hcy were extracted from the hitherto largest genome-wide association study (GWAS) with up to 44,147 individuals, and statistics data on association of SNPs with AF were obtained from another recently published GWAS with up to 1,030,836 individuals. SNPs were selected at a genome-wide significance threshold (*p* < 5 × 10^–8^). Fixed-effect inverse variance weighting (IVW) method was used to calculate the causal estimate. Other statistical methods and leave-one-out analysis were applied in the follow-up sensitivity analyses. MR-Egger intercept test was conducted to detect the potential directional pleiotropy.

**Results:**

In total, nine SNPs were identified as valid instrumental variables in our two-sample MR analysis. Fixed-effect IVW analysis indicated no evidence of causal association of genetically predicted Hcy with AF. The odds ratio (OR) and 95% confidence interval (CI) of AF per standard deviation (SD) increase in Hcy were 1.077 (0.993, 1.168), *p* = 0.075. Similar results were observed in the sensitivity analyses. MR-Egger intercept test suggested no evidence of potential horizonal pleiotropy.

**Conclusions:**

This two-sample MR analysis found no evidence to support causal association of Hcy with AF.

## Introduction

Atrial fibrillation (AF) is the most common sustained cardiac arrhythmia encountered clinically (Lip et al., 2012; Liu et al., 2018), and millions of individuals are expected to develop AF in the next decades (Violi et al., 2016). Patients with AF are at an increased risk of developing serious complications, including stroke, heart failure, dementia, and an early mortality (Magnani et al., 2011). Early detection and intervention for patients with AF can significantly reduce the risk of complications and improve survival rate (Ponikowski et al., 2016; Marrouche et al., 2018). Several risk factors for AF have been so far identified (Magnani et al., 2011).

As a well-known marker for pro-oxidation and proinflammation, elevated levels of homocysteine (Hcy) are considered as a risk factor/biomarker/predictor for developing cardiovascular disease (CVD) (Ganguly and Alam, 2015). In addition, several randomized controlled trials have suggested protective effect of lowering plasma Hcy levels with vitamin B supplements on overall CVD risk (Qin et al., 2016; Ahmed et al., 2019). However, only several studies have addressed the association between Hcy and AF risk (Schnabel et al., 2010). Although these studies have reported an association of elevated plasma Hcy levels with increased AF risk, it is difficult to distinguish causal association from spurious association in observational studies (Snezhitsky et al., 2016; Wei et al., 2017; Yao et al., 2017a). Further studies are warranted to clarify the causative relationship between Hcy and AF.

Mendelian randomization (MR) is an epidemiological approach for making causal inferences from observational data by using genetic variants as instrumental variables (Davey Smith and Hemani, 2014; Davies et al., 2018). Compared with classical observational studies, MR can effectively avoid biased associations because of confounding or reverse causality (Sekula et al., 2016). In this study, we aimed to investigate the causality between plasma Hcy levels and AF risk.

## Materials and Methods

### Study Design

**FIGURE 1:**
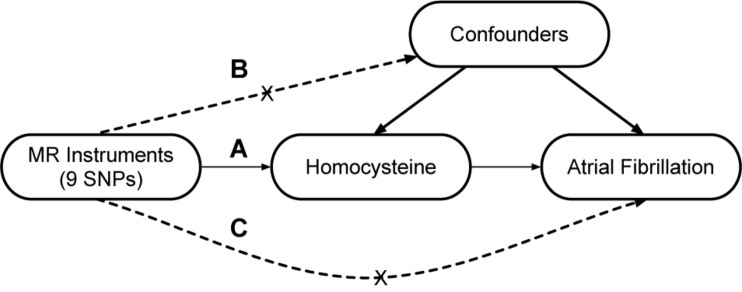
Design of the two-sample Mendelian randomization study. Three core assumptions were as follows: **(A)** the SNPs should be strongly associated with homocysteine; **(B)** the SNPs should not be related to confounders; **(C)** the SNPs should not be directly associated with atrial fibrillation.

A two-sample MR study was designed to explore the causal association of Hcy with AF. The single nucleotide polymorphisms (SNPs) that were selected as instrumental variables (IVs) were expected to fulfill three core assumptions (Figure 1; Sekula et al., 2016). First, the identified SNPs must be strongly associated with Hcy. Second, SNPs should not be related to confounders. Third, SNPs were only associated with AF through Hcy; in other words, SNPs should not be directly associated with AF.

### Data Sources

The exposure in this study was genetically predicted plasma Hcy levels. The genetic associations with Hcy were extracted from the hitherto largest genome-wide association study (GWAS) meta-analysis on Hcy. This meta-analysis included data from a total of 10 independent cohorts of European ancestry, with up to 44,147 individuals (Table 1; van Meurs et al., 2013).

**TABLE 1:** Overview of the datasets used for analyses.

**Exposure/outcome (data source)**	**Contributing studies (van Meurs et al., 2013; Nielsen et al., 2018)**	**Ancestry**	**Sample size (cases/controls)**	**Phenotyping**
Homocysteine (Meta-analysis of GWAS for homocysteine)	The CoLaus cohort	European	5,434	LC-MS/MS
	Rotterdam Study I (RSI)	European	3,414	LC-MS/MS
	Rotterdam Study II (RSII)	European	2,122	LC-MS/MS
	Nijmegen Biomedical Study (NBS)	European	550	LC-MS
	The TwinsUK cohort I (TUK I)	European	1,601	LC-MS/MS
	The TwinsUK cohort II (TUK II)	European	549	HPLC
	The InCHIANTI study	European	1,208	HPLC
	Women’s Genome Health Study (WGHS)	European	23,294	Enzymatic
	Framingham Heart Study (FHS)	European	3,105	HPLC
	Cardiovascular Health Study (CHS)	European	574	HPLC
	Baltimore Longitudinal Study of Aging (BLSA)	European	638	HPLC
	Nurses’ Health Study (NHS)	European	1,658	HPLC
Atrial fibrillation (meta-analysis of GWAS for atrial fibrillation)	The Nord–Trøndelag Health Study (HUNT)	European	69,635 (6,493/63,142)	ICD-9 427.3 or ICD-10 I48
	deCODE	European	371,632 (13,471/358,161)	ICD-9 427.3 or ICD-10 I48
	the Michigan Genomics Initiative (MGI)	European	12,275 (1226/11,049)	ICD-9 427.31
	DiscovEHR	European	48,482 (6,679/41,803)	ICD-10 I48
	United Kingdom Biobank	European	395,739 (14,820/380,919)	ICD-9 427.3 or ICD-10 I48
	2017 AFgen Consortium (Supplementary Table 1)	90% European	133,073 (17,931/115,142)	ICD-9, ICD-10, or 12-lead ECG

*LC-MS/MS, liquid chromatography–tandem mass spectrometry; LC-MS, liquid chromatography–mass spectrometry; HPLC, high-performance liquid chromatography; ICD, international classification of diseases; ECG, electrocardiogram.*

The outcome of this study was the lifetime risk of AF. The summary statistics data for AF were obtained from the recently published largest GWAS. This GWAS tested the associations between up to 34,740,186 genotyped SNPs and AF, including a total of 1,030,836 individuals (60,620 cases and 970,216 controls) of European ancestry from six resources [The Nord-Trøndelag Health Study (HUNT), deCODE, the Michigan Genomics Initiative (MGI), DiscovEHR, United Kingdom Biobank, and the AFGen Consortium] (Table 1 and Supplementary Table 1; Nielsen et al., 2018).

Studies contributing data to these GWAS meta-analyses had received ethical approval from relevant institutional review boards. In the present study, we only made use of the summarized data from these studies; hence, no additional ethics approval was required.

### SNPs Selection

To ensure a strong association between IVs and plasma Hcy levels, we selected SNPs associated with Hcy at a genome-wide significance threshold (*p* < 5 × 10^–8^) from the corresponding dataset. Then, we used LD-Link based on European to check for the independence of selected SNPs by calculating the pairwise-linkage disequilibrium (LD) (Machiela and Chanock, 2015; Myers et al., 2020). When *r*^2^ > 0.001, the SNP correlated with more SNPs or with higher *p* value dropped. In addition, we looked up the selected SNPs on PhenoScanner to evaluate whether these SNPs were associated with other traits (Staley et al., 2016). We performed an additional exclusion of SNPs associated with confounding traits at genome-wide significance level, which may affect the results. Subsequently, we extracted the genetic associations with the remaining SNPs and AF from the GWAS dataset on AF. When the specified SNP was not available in the AF dataset, a highly correlated SNP (*r*^2^ > 0.8) was selected for proxy. Any SNP directly associated with AF at genome-wide significance level was excluded. Finally, estimated variance in Hcy explained by each SNP and corresponding F statistics were calculated to evaluate the strength of IVs.

### Statistical Analysis

Fixed-effect inverse variance weighting (IVW) method was employed to estimate the causal effect of Hcy on AF. Specifically, Wald ratio was estimated for each SNP, and then, IVW mean of these ratio estimates was calculated as the effect estimate (Burgess et al., 2013). In the sensitivity analyses, the random-effect IVW, maximum likelihood, simple mode, weighted mode, simple median, weighted median, and MR-Egger methods were performed to test the robustness of the effect estimate. In addition, a leave-one-out sensitivity analysis was conducted to determine whether the result was affected by a single SNP (Burgess and Thompson, 2017). Subsequently, MR-Egger intercept test was applied to evaluate the potential horizontal pleiotropy, and funnel plot was generated to provide a visual inspection (Sterne et al., 2011; Bowden et al., 2015). Finally, mRnd was used to calculate the statistical power of the two-sample MR analysis, which required at least 80% (Freeman et al., 2013). The two-sided *p* < 0.05 was considered statistically significant. All the analyses were conducted using R packages (“MendelianRandomization” and “TwoSampleMR”) with R version 3.6.2 (Yavorska and Burgess, 2017; Hemani et al., 2018).

## Results

In total, 18 SNPs were extracted at a genome-wide significance threshold from the Hcy dataset. Among them, four SNPs (rs12921383, rs1801133, rs2851391, and rs957140) were dropped due to the linkage disequilibrium (Supplementary Table 2). Additional five SNPs (rs154657, rs2251468, rs548987, rs7422339, and rs9369898) were removed because of their associations with confounding traits. Specifically, rs154657 was associated with hypertension; rs2251468 was associated with cholesterol, C-reactive protein, and coronary artery disease; rs548987 was associated with body mass index; rs7422339 was associated with mass, cholesterol, and blood pressure; and rs9369898 was associated with cholesterol (Supplementary Table 3). After exclusion of these nine SNPs, the remaining nine SNPs were identified as IVs in our two-sample analysis. All the nine SNPs were valid (*F* > 10), and their characteristics are shown in Table 2.

**TABLE 2:** The characteristics of nine SNPs and their genetic associations with Hcy and AF.

**SNP**	**Nearest gene**	**Chr**	**EA**	**OA**	**EAF**	**F**	**SNP-Hcy association**			**SNP-AF association**		
							**Beta**	**SE**	***p* value**	**Beta**	**SE**	***p* value**
rs12134663	MTHFR	1	C	A	0.2	145	0.101	0.011	2.54E−21	0.0109	0.0089	0.22
rs12780845	CUBN	10	A	G	0.65	56	0.0529	0.009	7.8E−10	−0.0013	0.0071	0.85
rs1801222	CUBN	10	A	G	0.34	41	0.0453	0.007	8.43E−10	0.0069	0.0069	0.32
rs2275565	MTR	1	G	T	0.79	43	0.0542	0.009	1.96E−10	0.0002	0.0081	0.98
rs234709	CBS	21	C	T	0.55	113	0.0718	0.007	3.9E−24	0.0079	0.0072	0.27
rs42648	GTPB10	7	G	A	0.6	33	0.0395	0.007	1.97E−08	−0.0015	0.0068	0.82
rs4660306	MMACHC	1	T	C	0.33	37	0.0435	0.007	2.33E−09	0.0047	0.007	0.50
rs7130284	NOX4	11	C	T	0.93	89	0.1242	0.013	1.88E−20	0.0035	0.0123	0.78
rs838133	FUT2	19	A	G	0.45	39	0.0422	0.007	7.48E−09	0.0085	0.0072	0.24

*SNP, single nucleotide polymorphism; Hcy, homocysteine; AF, atrial fibrillation; Chr, chromosome; EA, effect allele; OA, other allele; EAF, frequency of effect allele; SE, standard error.*

**FIGURE 2:**
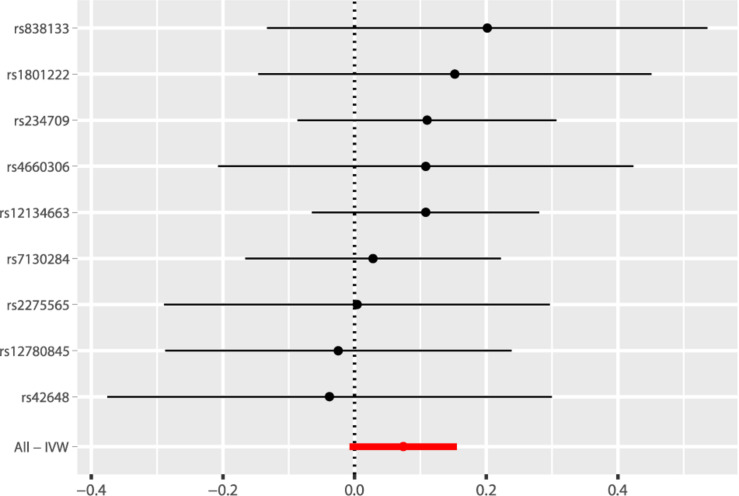
Fixed-effect IVW analysis of the causal association of Hcy with AF. The black dots and bars indicated the causal estimate and 95% CI using each SNP. The red dot and bar indicated the overall estimate and 95% CI meta-analyzed by fixed-effect IVW method. IVW, inverse variance weighted; Hcy, homocysteine; AF, atrial fibrillation; CI, confidence interval; SNP, single nucleotide polymorphism.

**FIGURE 3:**
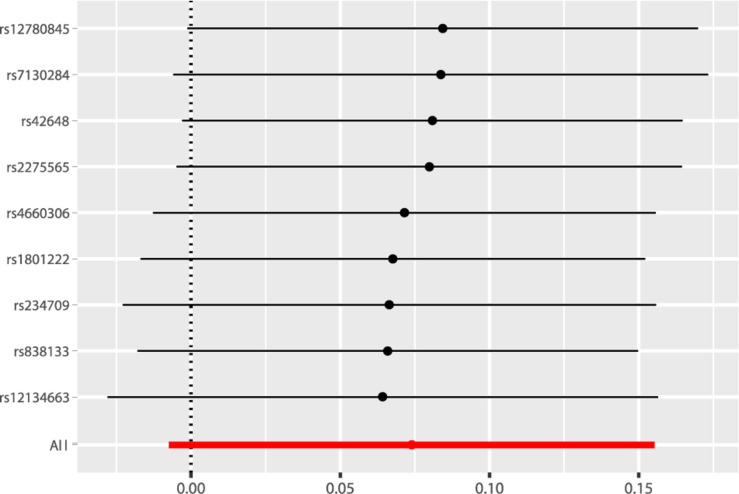
Leave-one-out analysis of the causal association of Hcy with AF. The black dots and bars indicated the causal estimate and 95% CI when a SNP was removed in turn. The red dot and bar indicated the overall estimate and 95% CI using fixed-effect IVW method. Hcy, homocysteine; AF, atrial fibrillation; CI, confidence interval; SNP, single nucleotide polymorphism; IVW, inverse variance weighted.

According to the fixed-effect IVW analysis results, the odds ratio (OR) and 95% confidence interval (CI) of AF per standard deviation (SD) increase in Hcy were 1.077 (0.993, 1.168), *p* = 0.075 (Figure 2, Table 3, and Supplementary Figure 1). These results suggested that genetically predicted plasma Hcy levels were not associated with AF. Similar results were observed using other statistical methods (Table 3). The leave-one-out analysis results indicated that the overall estimate was not driven by any SNP (Figure 3). According to the MR-Egger intercept test results, the overall estimate and 95% CI of the intercept were 0.000 (−0.013, 0.014), *p* = 0.958 (Table 4). Together with the almost symmetrical funnel plot (Supplementary Figure 2), these results suggested no evidence of horizonal pleiotropy. In addition, no significant association between Hcy and HF was observed in MR analyses that included nine SNPs dropped due to linkage disequilibrium or potential pleiotropy (Supplementary Table 4).

**TABLE 3:** Association of plasma Hcy levels with AF risk using various methods.

**Method**	**OR**	**LCI**	**UCI**	***p*-value**
Fixed-effect IVW	1.077	0.993	1.168	0.075
Random-effect IVW	1.077	0.993	1.168	0.075
Maximum likelihood	1.077	0.993	1.169	0.074
Simple mode	1.121	0.966	1.301	0.171
Weighted mode	1.108	0.970	1.266	0.169
Simple median	1.114	0.999	1.242	0.052
Weighted median	1.106	0.998	1.226	0.054
MR-Egger	1.071	0.856	1.340	0.551

*Hcy, homocysteine; AF, atrial fibrillation; OR, odds ratio; LCI, lower confidence interval; UCI, upper confidence interval; IVW, inverse variance-weighted.*

**TABLE 4:** MR-Egger intercept test results of association between Hcy and AF.

**Estimate**	**Standard Error**	**LCI**	**UCI**	***p* value**
0.000	0.007	−0.013	0.014	0.958

*MR, Mendelian randomization; Hcy, homocysteine; AF, atrial fibrillation; LCI, lower confidence interval; UCI, upper confidence interval.*

## Discussion

This is a two-sample MR study to investigate the association between plasma Hcy levels and the risk of AF. In the present study, there is no evidence to support causal association of Hcy with AF. It should be cautious, since the observed *p* value of 0.075 in the primary analysis might be suggestive of a causal relationship and all of the secondary analyses point to a consistent effect direction. However, the MR analyses that used all extracted SNPs also suggested insignificant association between Hcy and AF.

Homocysteine is an intermediate product of methionine metabolism through a series of transmethylation reactions. Hcy-induced oxidative stress and impairments in methylation activity have been thought to play an important role in the pathogenesis of CVD (Ullegaddi et al., 2004; Shen et al., 2010). Previous studies have suggested that elevated plasma Hcy levels (known as hyperhomocysteinemia) were associated with an increased risk of CVD including hypertension (Kim et al., 2018) and coronary artery disease (Jakubowski, 2019), which were potential risks for AF. Increased Hcy confers an independent risk for the thromboembolic and cardiovascular events in AF patients (Yao et al., 2017b). Cross-sectional clinical studies indicated that AF patients had elevated Hcy levels, especially in elderly patients (Marcucci et al., 2004; Shimano et al., 2008). Several observational clinical studies have reported the positive association of Hcy with recurrent AF risk after ablation (Yao et al., 2017b). However, the results of this study about the effects of Hcy on AF were different from those of previous studies. One of the possible reasons is that the sample size might not be large enough in traditional observational studies to detect the exact association. Moreover, confounding factors are inevitable in observational studies, which can cause bias. Hcy may be just a sign of other CVDs, which in turn increased AF risk. Additionally, high plasma Hcy levels observed in traditional studies might also be a consequence of the AF incident.

Observational studies often suffer from confounding factors that can lead to spurious non-replicable findings (Lawlor et al., 2008). Randomized controlled trials (RCTs) are the gold standard to establish causal relationships in the medical research (Evans and Davey Smith, 2015). However, RCTs cannot always be conducted because they can be excessively costly, impractical, even unethical, or difficult to collect a large enough sample (Lawlor et al., 2008; Evans and Davey Smith, 2015). One of the alternative approaches is conducting MR experiments that are based on Mendel’s law of independent assortment, i.e., each trait inherits independently from other traits to the next generation. In MR, the random segregation of alleles (genes) helps in dividing them into exposed and control groups independently, and unmeasured confounders are also equally distributed between two groups (Gupta et al., 2017). The application of MR can ingeniously remedy the shortcomings of traditional epidemiological research, such as confounding factors and reverse causation, providing method for epidemiological research with regard to etiology (Badsha and Fu, 2019). MR studies select suitable genetic instrumental variables from the widely available GWAS, which make it a time- and cost-efficient approach (Holmes et al., 2017). These advantages contribute to its increasing popularity for assessing and screening for potentially causal associations. In this study, we analyzed the association between plasma Hcy levels and AF with the aid of a large-scale GWAS. This study suggested that an increase in plasma Hcy levels did not directly lead to the occurrence of AF. As shown in the research, several recent prospective studies have shown that Hcy might be a novel risk marker for AF rather than a causal risk factor (Schnabel et al., 2010; Kubota et al., 2019). These findings suggested that the possibility of Hcy as an independent risk factor in AF patients was small.

Strengths of the present study include the two-sample MR study design and the large sample size. The following potential limitations also require discussion. First, it is difficult to completely rule out the influence of potential directional pleiotropy, which may lead to biased estimates. However, SNPs associated with known confounding traits were excluded in our analyses. In addition, no evidence of pleiotropic effect was observed in MR-Egger intercept test, and similar results were observed in sensitivity analyses using several other models. Second, there is some degree of overlap between the participants included in the GWAS for Hcy and AF, which can cause biased estimate if substantial. Five cohorts included in the Hcy GWAS are also part of the AF GWAS (3.5%), although true proportions might be smaller. Therefore, risk of bias from sample overlap is probably low. Third, we are unable to perform the reverse analysis because the GWAS for Hcy is not publicly available. Finally, we only reveal the relationship between Hcy and AF from a genetic point of view, without involving other environmental factors.

## Conclusion

This two-sample MR analysis found no evidence to support causal association of Hcy with AF. It was plausible that simply reducing plasma Hcy levels could not decrease the incidence of AF in clinical practice. However, additional studies are still needed to further confirm our results.

## Data Availability Statement

The datasets presented in this study can be found in online repositories. The names of the repository/repositories and accession number(s) can be found in the article/Supplementary Material.

## Author Contributions

SC and WZ designed the study. SC, FY, and TX conducted the analysis and drafted the first draft. YW, KZ, and GF reviewed the design and analysis, commented on the text, and provided subject matter expertise. All authors reviewed the final version for intellectual content.

## Conflict of Interest

The authors declare that the research was conducted in the absence of any commercial or financial relationships that could be construed as a potential conflict of interest.
