# Supplementary material for: Appraising the Causal Association of Plasma Homocysteine Levels With Atrial Fibrillation Risk: A Two-Sample Mendelian Randomization Study
Source: Front Genet. 2021 May 26;12:619536. doi: 10.3389/fgene.2021.619536 (PMC8189424; doi:10.3389/fgene.2021.619536)
Supplement: Supplementary file 1 [file Data_Sheet_1.docx]

**SUPPLEMENTAL MATERIAL**

**Supplemental tables**

**Table S1.** Studies contributing to 2017 AFgen Consortium.

| Study | Ancestry group | Sample size | Cases | Controls |
| --- | --- | --- | --- | --- |
| Atrial Fibrillation Biobank LMU/Cooperative Health Research in the Region of Augsburg (AFLMU/KORA) | European | 886 | 448 | 438 |
| Age, Gene/Environment Susceptibility Study (AGES) Reykjavik study | European | 2893 | 785 | 2108 |
| Angiography and Genes Study (ANGES) | European | 779 | 226 | 553 |
| Atherosclerosis Risk in Communities Study (ARIC) | African American | 2768 | 278 | 2490 |
| Atherosclerosis Risk in Communities Study (ARIC) | European | 8880 | 1420 | 7460 |
| Basel Atrial Fibrillation Cohort Study (BEAT-AF) | European | 3040 | 1520 | 1520 |
| Biobank Japan | East Asian | 4130 | 837 | 3293 |
| Cleveland Clinic Lone Atrial Fibrillation GeneBank Study (CCAF) | European | 3536 | 606 | 2930 |
| Cardiovascular Health Study (CHS) | African American | 801 | 189 | 612 |
| Cardiovascular Health Study (CHS) | European | 3201 | 1011 | 2190 |
| COROGENE | European | 2226 | 248 | 1978 |
| Framingham Heart Study (FHS) | European | 4404 | 880 | 3524 |
| Finnish Cardiovascular Study (FINCAVAS) | European | 2879 | 971 | 1908 |
| Generation Scotland: Scottish Family Health Study (GS:SFHS) | European | 6854 | 203 | 6651 |
| Ludwigshafen Risk and Cardiovascular Health (LURIC) | European | 3034 | 368 | 2666 |
| Malmö Diet and Cancer study (MDCS) | European | 5877 | 1232 | 4645 |
| Mount Sinai BioMe Biobank | African American | 2306 | 174 | 2132 |
| Mount Sinai BioMe Biobank | European | 1151 | 291 | 860 |
| Mount Sinai BioMe Biobank | Hispanic | 3358 | 277 | 3081 |
| Multi-Ethnic Study of Atherosclerosis (MESA) | European | 2527 | 155 | 2372 |
| Massachusetts General Hospital Atrial Fibrillation study (MGH AF) | European | 1277 | 366 | 911 |
| Massachusetts General Hospital Cardiology and Metabolic Patient cohort (MGH CAMP) | European | 2793 | 665 | 2128 |
| Prospective Investigation of Vasculature in Uppsala Seniors (PIVUS) | European | 949 | 154 | 795 |
| Prevention of Renal and Vascular Endstage Disease (PREVEND) | European | 3520 | 113 | 3407 |
| PROspective Study of Pravastatin in the Elderly at Risk (PROSPER) | European | 5244 | 505 | 4739 |
| Rotterdam Study I (RS I) | European | 5947 | 1025 | 4922 |
| Rotterdam Study II (RS II) | European | 1806 | 146 | 1660 |
| Rotterdam Study III (RS III) | European | 3030 | 121 | 2909 |
| Study of Health in Pomerania (SHIP) | European | 1921 | 106 | 1815 |
| Sao Paolo Heart Failure Cohort (SPHFC) | Brazilian | 955 | 197 | 758 |
| Swedish Twin Registry (TWINGENE) | European | 6813 | 403 | 6410 |
| Uppsala Longitudinal Study of Adult Men (ULSAM) | European | 1120 | 294 | 826 |
| Vanderbilt University Medical Center BioVU Biorepository | European | 10185 | 428 | 9757 |
| Wellcome Trust Case Control Consortium 2 Munich (WTCCC2-Munich) | European | 1127 | 330 | 797 |
| Women’s Genome Health Study (WGHS) | European | 20856 | 959 | 19897 |

**Table S2.** The R^2^ of 4 SNPs dropped due to linkage disequilibrium.

| RS1 | RS2 | R^2^ |
| --- | --- | --- |
| rs1801133 | rs12134663 | 0.1277 |
| rs957140 | rs7130284 | 0.0641 |
| rs12921383 | rs154657 | 0.0701 |
| rs2851391 | rs234709 | 0.0201 |

**Table S3.** Association of 5 SNPs with confounding traits at a genome-wide significance level (P < 5×10^-8^).

| SNP | Nearest gene | Chr | EA | OA | EAF | Confounding traits |
| --- | --- | --- | --- | --- | --- | --- |
| rs154657 | DPEP1 | 16 | A | G | 0.47 | High blood pressure, Hemoglobin concentration |
| rs2251468 | HNF1A | 12 | C | A | 0.35 | Low density lipoprotein, Coronary artery disease, Total cholesterol, High cholesterol, C-reactive protein |
| rs548987 | SLC17A3 | 6 | C | G | 0.13 | Body mass index |
| rs7422339 | CPS1 | 2 | A | C | 0.33 | Fat-free mass, Weight, High density lipoprotein, Systolic blood pressure, Chronic kidney disease, Hip circumference |
| rs9369898 | MUT | 6 | A | G | 0.62 | Total cholesterol |

SNP, single nucleotide polymorphism; Chr, chromosome; EA, effect allele; OA, other allele; EAF, frequency of effect allele.

**Table S4.** MR analyses of association between Hcy and AF that used all extracted SNPs.

| Method | OR | LCI | UCL | p-value |
| --- | --- | --- | --- | --- |
| Fixed-effect IVW | 0.995 | 0.947 | 1.045 | 0.838 |
| Random-effect IVW | 0.995 | 0.945 | 1.047 | 0.844 |
| Maximum likelihood | 0.995 | 0.947 | 1.045 | 0.842 |
| Simple mode | 1.030 | 0.891 | 1.189 | 0.696 |
| Weighted mode | 0.980 | 0.900 | 1.066 | 0.641 |
| Simple median | 1.029 | 0.946 | 1.118 | 0.508 |
| Weighted median | 0.963 | 0.894 | 1.036 | 0.313 |
| MR-Egger | 0.929 | 0.830 | 1.039 | 0.216 |

MR, Mendelian randomization; Hcy, homocysteine; AF, atrial fibrillation; OR, odds ratio; LCI, lower confidence interval; UCI, upper confidence interval; IVW, inverse variance-weighted.

**Supplemental figures**

**Figure S1.** Scatter plot of the association of each SNP with Hcy and AF. The dots and bars indicated the causal estimate and 95% CI using each SNP. The line indicated the association of Hcy and AF analyzed using fixed-effect IVW. SNP, single nucleotide polymorphism; Hcy, homocysteine; AF, atrial fibrillation; CI, confidence interval; IVW, inverse variance-weighted.

**Figure S2.** Funnel plot of the MR analysis for causal association of Hcy with AF. X axis presented the causal estimates and Y axis presented the inverse SE. The dots indicated each SNP, and the line indicated the overall estimate using fixed-effect IVW method. MR, Mendelian randomization; Hcy, homocysteine; AF, atrial fibrillation; SE, standard error; SNP, single nucleotide polymorphism; IVW, inverse variance-weighted.

**Figure S1.**

**Figure S2.**
